# Ethical concerns on sharing genomic data including patients’ family members

**DOI:** 10.1186/s12910-018-0310-5

**Published:** 2018-06-18

**Authors:** Kyoko Takashima, Yuichi Maru, Seiichi Mori, Hiroyuki Mano, Tetsuo Noda, Kaori Muto

**Affiliations:** 1 Department of Public Policy, The Institute of Medical Science, The University of Tokyo, Shirokanedai 4-6-1, Minato-ku, Tokyo, 108-8639 Japan; 2 Medical Genomics Center, National Center for Global Health and Medicine, 1-21-1 Toyama, Shinjuku-ku, Tokyo, 162-8655 Japan; 3 Department of Regional Policy, Faculty of Regional Sciences, Tottori University, 4-101 Koyama-cho Minami, Tottori, 680-8551 Japan; 4 Cancer Precision Medicine Center, Japanese Foundation for Cancer Research, 3-8-31, Ariake, Koto-ku, Tokyo, 135-8550 Japan; 5 National Cancer Center Research Institute, 5-1-1 Tsukiji, Chuo-ku, Tokyo, 104-0045 Japan; 6 Cancer Institute, Japanese Foundation for Cancer Research, 3-8-31, Ariake, Koto-ku, Tokyo, 135-8550 Japan

**Keywords:** Genomic research, Data sharing, Human research protection, Patients and family members, ELSI

## Abstract

**Background:**

Platforms for sharing genomic and phenotype data have been developed to promote genomic research, while maximizing the utility of existing datasets and minimizing the burden on participants. The value of genomic analysis of trios or family members has increased, especially in rare diseases and cancers. This article aims to argue the necessity of protection when sharing data from both patients and family members.

**Main text:**

Sharing patients’ and family members’ data collectively raises an ethical tension between the value of datasets and the rights of participants, and increases the risk of re-identification. However, current data-sharing policies have no specific safeguards or provisions for familial data sharing. A quantitative survey conducted on 10,881 general adults in Japan indicated that they expected stronger protection mechanisms when their family members’ clinical and/or genomic data were shared together, as compared to when only their data were shared. A framework that respects decision-making and the right of withdrawal of participants, including family members, along with ensuring usefulness and security of data is needed. To enable this, we propose recommendations on ancillary safeguards for familial data sharing according to the stakeholders, namely, initial researchers, genomic researchers, data submitters, database operators, institutional review boards, and the public and participants.

**Conclusions:**

Families have played significant roles in genetic research, and its value is re-illuminated in the era of genomic medicine. It is important to make progress in data sharing while simultaneously protecting the privacy and interests of patients and families, and return its benefits to them.

## Background

### A new scientific standard: data sharing

The idea that data sharing promotes scientific progress has been widely accepted among research communities, funding bodies, and regulatory agencies [1–5]. Sharing clinical and genomic data promises to increase research efficiency, expedite translational efforts of research results, and ensure the traceability and transparency of published studies, while maximizing the utility of existing datasets and minimizing the burden to participants. Highly shared data sets include genomic research for cancer and rare diseases. Global sharing platforms have been developed, such as the International Cancer Genome Consortium (ICGC) [6], the Global Alliance for Genomic Health (GA4GH) [7], and the International Rare Diseases Research Consortium (IRDiRC) [8]. In genomic research for cancer and rare diseases, patient family members (e.g., parents, children, siblings, or uncles/aunts) can provide important information [9–11]. In some studies, genomic data from patients and family members are routinely submitted to a repository or database, and shared broadly. While the advantage of genomic data sharing is recognized, privacy protection, security, and respecting participants’ consent remain as ethical concerns [2, 12, 13]. Do these concerns differ between sharing only patient data and those from both them and family members? We argue that, when sharing clinical and genomic data, there is a need to consider protection not only of the patients’ own data but also their family members’ data. The goal of this article is to suggest roles of stakeholders including genomic researchers, data submitters, database operators, institutional review boards, and the public and participants, for building the proper balance between sharing data to expedite genomic research discoveries and protecting patients’ and families’ privacy and interests.

## Main text

### Current data-sharing policies of databases and academic journals

Governments, scientific repositories and databases, and academic journals have established data-sharing policies to protect individual participants [5]. The US National Institutes of Health (NIH) employs a “Genomic Data Sharing Policy”, which requires all NIH-funded research investigators to submit large-scale human genomic data and relevant associated data to an NIH-designated data repository [14]. The policy requires submitting institutions and institutional review boards (IRBs) to determine the appropriateness of submitting and sharing data. Investigators are required to obtain informed consent from participants for future research purposes and their data-sharing plan, including whether individual-level data are shared. Similarly, in Japan, data from all nationally supported research must be deposited in the National Bioscience Database Center (NBDC) or another international database, following the NBDC’s guidelines for accepting submitted data [15]. However, neither guideline employs specific safeguards or provisions for sharing family members’ data together with patients’ data.

We previously investigated publication policies on data availability of major journals and publishing groups and found that most major academic journals mandate data sharing, in that researchers must submit their study data to a public repository or database and provide relevant accession numbers [16]. Some journals even explicitly state that the unavailability of such data is a reason for rejection. This trend is supported based on research integrity (i.e., transparency, traceability, and accountability) and the scientific obligation of maximizing efficiency and effectiveness [17, 18]. According to our investigation results, while most policies factor in consent and privacy protection for patients/participants, no standardized rules for sharing family members’ data are in use [16]. In fact, several genomic studies on familial diseases, including patients and their family members as participants, provide sequence data in the database and family trees in the published articles [19–21]. As the risk of identifiability or harm for participants in genomic data sharing varies among different datasets, data-sharing policies should be arranged according to the sensitiveness of the datasets [5]. Thus, if sharing of data from patients and families, or familial data sharing, carries higher risks of identifiability or harm, present data sharing policies are not enough to protect participants including patients’ family members.

### Concerns for sharing family-member genomic data with patient data

Combining genomic data from patients and family members is valuable because of its comprehensiveness, although this requires researchers to obtain informed consent for data sharing from all participants who contribute to the dataset (i.e., patients and close family members). Thus, some participants might not be able to make their decision freely when under implicit or explicit pressure from researchers or other enrolled family members, even if they are hesitant regarding their data being shared. For genomic researchers, using participants’ data for their study and submitting those data to a public database are standard aspects of the research, but for participants, allowing their clinical and genomic data to be studied and agreeing that the data be shared publically may raise other issues.

Another issue is ensuring the participants’ right to withdraw from the research. This right is one of the fundamental principles of medical research ethics, but is extremely difficult to achieve with international data sharing when data and samples are shared widely [22]. While data from patients and family members facilitate cancer genomic research, withdrawal of some family members might limit the overall value. However, it is unacceptable when individual participants (including family members) feel it is difficult to express their wish to discontinue the use and sharing of their personal data.

Recent data suggested that coded or “anonymized” sequenced DNA can be linked to individuals as genetic databases proliferate [23–26]. There are two possible vulnerabilities that increase the risk of re-identification when submitting genomic data of patients and family members to a repository or database. First, when results from familial or trio genomic analysis are submitted, those data are usually shared with clinical data for patients and family members to enable analysis of genomic data and phenotypes. A dataset is usually associated with the published article showing these data, or its accession number is shown. These articles often include some (but not all) family pedigrees. A second vulnerability arises from the rarity of the targeted disease. Hereditary cancers are thought to comprise only 5–10% of all cancers [27]. Hence, studies including relatively small number of families can be published when they have scientific value, which is why data sharing or a registry is truly important in this research area. Both vulnerabilities raise the ethical concern of re-identifying research participants. For example, we found an article involving three families with the same familial hematological cancer that identified the recruiting hospitals, mentioned that informed consent was obtained, showed the pedigrees of three families (i.e., revealing the number of brothers, sisters, and children in each family), and the accession code [19]. Those genomic data were submitted as controlled-access data, and a secondary user would need permission to access the data. This process is appropriate under the current rules mentioned above. However, viewed from the perspective of participants, the possibility of re-identification seems higher than with non-familial or singleton studies. This may rarely happen, but researchers have a responsibility to lower risks that can harm or violate participants’ rights and interests.

These concerns should be considered when protecting and honoring research participants. The question naturally arises as to what opinion the public or potential research participants have regarding these issues.

### Public high expectations for rigorous protection of familial data sharing

Some data indicate that the public recognizes the value and benefits of data sharing [28, 29]. Other findings indicate that participants tend to trust academic researchers or non-profit organizations, but are reluctant to share data with commercial affiliations. However, almost all studies focused only on sharing participants’ own data, without considering sharing family members’ data. To understand public attitudes, we conducted an online survey of Japanese adults through a monitor panel of a marketing research company (INTAGE Inc.). The targeted population was 44,360 men and women aged 20–60 years who were chosen based on Japan’s population distribution and age structure. The survey was sent and responses were collected during February 17–20 in 2017. There were 10,881 respondents (response rate: 24.5%), included 5397 men (49.6%) and 5484 women. When participants were asked “which one do you think needs the strongest protection for data sharing?” with a brief explanation of data sharing, the most common answer (39.3%) was that protection should be strengthened when their family members’ data were also shared, versus only their own clinical and/or genomic data. Additionally, participating patients (those who went to a hospital or were hospitalized during the last year) tended to consider that sharing family members’ data needs more rigorous safeguards, when compared with participants who did not visit hospitals (Table 1). This is a single study result, and further studies are required for reflecting patients and public opinion into specific conclusion. However, the results indicate that there are public concerns (especially among patients) about the potential for greater risks when sharing their own data with family members’ clinical and genomic data. Hence, unless researchers take additional measures, beyond general privacy protection, research participants (including family members) may consent to share their samples and data without full appreciation of the risks involved.

**Table 1:** Attitudes of healthy adults and patients against sharing clinical and genomic data including family members

Which one do you think needs the strongest protection for data sharing?	Healthy adults (%)	Patients (%)	Total general adults (%)
Only your clinical data (i.e., name of diagnosed disease, medical history)	478 (8.9)	455 (8.3)	933 (8.6)
Your clinical data and your genomic data	555 (10.3)	672 (12.2)	1227 (11.3)
Your clinical data, your genomic data, and your relatives’ clinical and genomic data	1802 (33.4)	2479 (45.1)	4281 (39.3)
No difference in these three for necessary protection	1094 (20.3)	1160 (21.1)	2254 (20.7)
I do not know/I cannot answer	1460 (27.1)	726 (13.2)	2186 (20.1)
Total	5389 (100)	5492 (100)	10,881 (100)

### Recommendations: safeguard for familial data sharing

Authors, a group of genomic researchers, a sociologist, a bioethicist and a philosopher of law, shared these concerns about the necessity of specific protection in family genomic data sharing, and discussed how to protect patients and family members whose genomic data are submitted to a repository and shared broadly. After several discussions, we reached a consensus, and here we offer recommendations below for how familial genomic data should be shared, according to the roles and responsibilities of each stakeholder. We do not think these are conclusively the best measures; however, we hope this article will engage not only the scientific community but also the public to discuss the value and vulnerability of families in genomic data sharing.

#### Initial researchers with whom participants offer their data or specimens

Before obtaining informed consent from participants, researchers should explain not only general considerations for submitting and sharing their clinical and genomic data, but also how their data will be presented in databases or publications (especially regarding the relationship between family members, i.e., child, parent, sibling, aunt/uncle, etc.) and what measures will be taken to protect them. Informed consent should be obtained from all participants (patients and family members), without any pressures, even from enrolled family members when family-based recruitment approach is applied [30]. If some family members provide consent for participating in the research, but refuse to share their data, this wish must be honored even if the value of the dataset is weakened. If researchers use specimens and data obtained without prior intention for submitting it to a repository or database, then we propose that approval from an IRB alone is insufficient for sharing data from family members. Instead, researchers should re-contact the participants to ask whether their genomic and clinical data can be shared. Re-contacting research participants presents practical difficulties, especially when data came from past studies and the participants may have died or moved, and researchers do not have current information to contact them. However, in familial genomic data sharing, researchers should try to confirm participants’ wishes for data sharing because of the vulnerabilities of families, as discussed above. As a prospective strategy, an effective approach is to adopt an e-consent system, such as dynamic consent [5, 22], so that participants can watch how their resources are used and can express their will continuously, as an increased risk of re-identification can affect participants’ decisions-making whether to continue in the study and allow researchers to share their data. The most important consideration is offering an opportunity for participants or family members to withdraw from additional use of their specimens and data before they are distributed.

#### Data submitters

In some cases, initial researchers and data submitters are the same individuals, but they might be different. Genomic researchers should recognize the added risks of sharing patient and family-member data collectively. Risk considerations should be reflected in the data-sharing plan. Most database have tiered levels of accessibility: “open-access data” or “uncontrolled-access data” (available without any restrictions) and “controlled-access data” (available for users meeting specific requirements including an approval from a data-access committee). Patient and family-member data should be submitted as “controlled-access data” to ensure that secondary users are reviewed in terms of the relevance of their research aims and experience, and whether the security level of the system in their institutions is adequate.

Data submitters should also take measures to lower the risk of re-identification by (for example) not adding identifiable information (e.g., the age, sex, and relationship of each patient’s relative) to a database initially. Potential data users who need those data could directly contact the repository or a researcher responsible for the dataset to establish a collaborative study under a confidentiality agreement.

#### Database operators

Researchers, governmental entities, and/or organizations involved in developing and operating databases and repositories should recognize the sensitive nature and possible risks of familial data sharing. Most operators have already prohibited data users from trying to re-identify individuals from datasets. This should be interpreted that attempting to re-identify or examine genetic relationships within a family using sequencing data combined with clinical and familial information from datasets beyond the original study purpose is also prohibited. Hopefully, databases and repositories will take remedial measures with small datasets. For example, databases could collect data in a temporarily closed location within the database for the same disease, releasing it only after findings from several studies have accumulated. This approach can decrease the risk of re-identification. If researchers require more secure operation, the repository could consider permitting temporary submission of pooled and scrambled data from several individuals. Then, secondary users could contact the submitting or responsible researcher for further data, so that the responsible researcher can know who is using the data and can manage the requirements that the research participants consented to, perhaps via a collaborative relationship. Operators should also regularly supervise whether submitted data are used under the required conditions. Moreover, for becoming more engaged, databases/repositories need to release information or education tools for the public about data sharing, genome, and the meaning of family members. We consider it is because 1) the database is almost a public infrastructure and has an accountability and 2) this is one of the ways of demonstrating respect for participants.

#### IRBs

Research ethics committees or IRBs also play important roles in protecting the privacy and interests of patients and family members. In the context of clinical trials, Doernberg and Wendler suggested that an IRB is responsible for ensuring the timely reporting of trial results because maximizing the benefit of research supports the respect of participants and “IRBs should mandate that individual patient data are shared” [31]. Although the situations of data sharing in clinical trials and genomic research are somewhat different, IRBs are considered to have the same responsibility for reviewing research based on ethical principles. When IRBs review genomic research involving familial data sharing (particularly in cancer and rare diseases), they should require researchers to show the data-sharing plan in their protocols. In the process of data submission, IRBs sometimes need to explain their policies to researchers or a repository. Furthermore, when researchers terminate their studies, IRBs should confirm whether the data will be submitted to a database as planned, or if any appropriate changes will be made.

#### Public and research participants

Finally, social and public understanding is necessary for scientific databases, especially for genomic research, where sensitive participant data will be shared broadly. Participants (including family members) and the public need to take interest in and oversee how human genomic and clinical data are used in research and shared.

## Conclusions

We are living in the era of individual genome sequencing, the ultimate source of “personal information”, and global sharing of such data. This phenomenon re-illuminates the value of families, which usually share partial genomic information and have different phenotypes, in biomedical research. However, the risk of re-identification of data contributors increases when sharing the clinical and genomic data of patients and family members. Our online survey showed that the public, especially recent patients, considered familial data sharing in research should be protected more strictly. It is important that all stakeholders, including not only researchers who initially collect specimens and data, but also data submitters, database operators, IRBs, and public and research participants, fulfill their own responsibilities to protect patients’ and their families’ privacy and interests while advancing genomic science and medicine. Further research on the opinions and attitudes of the public and patients is required and it is necessary to review current rules continuously as new techniques are developed and social values change.

## Abbreviations

IRB: Institutional review board
NBDC: National Bioscience Database Center (Japan)
NIH: National Institutes of Health (US)
